# Vocal complexity in a socially complex corvid: gradation, diversity and lack of common call repertoire in male rooks

**DOI:** 10.1098/rsos.231713

**Published:** 2024-01-10

**Authors:** Killian Martin, Francesca M. Cornero, Nicola S. Clayton, Olivier Adam, Nicolas Obin, Valérie Dufour

**Affiliations:** ^1^ PRC, UMR 7247, Ethologie Cognitive et Sociale, CNRS-IFCE-INRAE-Université de Tours, Strasbourg, France; ^2^ Department of Psychology, University of Cambridge, Cambridge, UK; ^3^ Institut Jean Le Rond d'Alembert, UMR 7190, CNRS-Sorbonne Université, 75005 Paris, France; ^4^ Institut des Neurosciences Paris-Saclay, UMR 9197, CNRS-Université Paris Sud, Orsay, France; ^5^ STMS Lab, IRCAM, CNRS-Sorbonne Université, Paris, France

**Keywords:** bioacoustics, bird, repertoire, heterogeneity, clustering

## Abstract

Vocal communication is widespread in animals, with vocal repertoires of varying complexity. The social complexity hypothesis predicts that species may need high vocal complexity to deal with complex social organization (e.g. have a variety of different interindividual relations). We quantified the vocal complexity of two geographically distant captive colonies of rooks, a corvid species with complex social organization and cognitive performances, but understudied vocal abilities. We quantified the diversity and gradation of their repertoire, as well as the inter-individual similarity at the vocal unit level. We found that males produced call units with lower diversity and gradation than females, while song units did not differ between sexes. Surprisingly, while females produced highly similar call repertoires, even between colonies, each individual male produced almost completely different call repertoires from any other individual. These findings question the way male rooks communicate with their social partners. We suggest that each male may actively seek to remain vocally distinct, which could be an asset in their frequently changing social environment. We conclude that inter-individual similarity, an understudied aspect of vocal repertoires, should also be considered as a measure of vocal complexity.

## 1. Introduction

Living in a complex social system imposes communicative demands on individuals, ranging from the exchange of a wide variety of information to discriminating between conspecifics and heterospecifics [1,2]. The social complexity hypothesis for communicative complexity posits that there should be a relationship between social and communicative complexity [3,4]. The social intelligence hypothesis [5,6] also posits that social species, including corvids, have acquired advanced cognitive skills to meet the demands of their sociality. Following both hypotheses, we can predict that social species with advanced cognitive aptitudes should also exhibit vocal complexity. Vocal complexity can be addressed by looking at several features of the repertoire of a species, including its diversity (e.g. the number of vocal units in the repertoire) and its flexibility [7]. Vocal flexibility can be either structural, such as with gradation, where vocalizations of different types exist at either end of a continuum, and vocalizations gradually shift in structure from one type to the other [7–9], or functional, such as when an individual produces acoustically identical vocalizations in several different contexts [10,11]. The notion of vocal flexibility is an intriguing concept. On the one hand, vocal flexibility should provide communicative advantages, as it potentially allows the transmission of more complex signals, with more nuances and expressivity [7]. On the other hand, vocalizing has the main communicative function of conveying a message to conspecifics. This is in general true of the single calls in the repertoire (hereafter referred to as call units), which serve functions as diverse as warning about predators, signalling food location, making social interactions or coordinating social activities [1,2]. To remain intelligible to listeners, call units should remain somewhat standardized in both acoustic and functional terms.

Aside from call units, the potential for vocal flexibility also exists in the songs of adult birds. Songs are composed of vocal units (hereafter referred to as song units), which can be reused call units or entirely different vocalizations. In songs, different structural arrangements of song units appear functionally equivalent, for example serving reproductive or territorial functions [12,13]. This may allow greater freedom for different individuals to flexibly produce different songs, either through variable song unit choice, or through variable sequential organization. However, the vast majority of bird species sing the same stereotyped song throughout their life after acquiring their songs as juveniles [14]. In extreme cases, an individual may produce only a single stereotyped song, as in the zebra finch *Taeniopygia guttata* [15,16] or the white-crowned sparrow *Zonotrichia leucophrys* [17]. Only comparatively few species, called open-ended learners, appear able to modify their song past the juvenile acquisition phase [18]. Vocal flexibility may thus lead to varying degrees of inter-individual similarity.

Aside from the diversity and flexibility of the repertoire, other inter-individual vocal variations may be markers of social complexity. One example is the phenomenon called ‘vocal signature’ [19–21], which does not impact function, but can serve to identify individuals (e.g. in birds [22–27]; primates [28–32]; mongoose [33]; cetaceans [34–36]), or can signal social group membership [36,37]. Another example are dialects, variations between geographically separate populations [12,38,39]. Dialects may be a by-product of vocal learning, as many species acquire their vocalizations by learning from social partners [12] such as parents and affiliated individuals. Given their communicative function, the vocalizations of different individuals or groups are not expected to greatly deviate from what is typical in the species' repertoire in terms of their acoustic structure, as it may otherwise hinder transmission of information to receivers of the vocalization.

Birds, with their diverse aggregation patterns, are ideal species to study the link between social systems and communicative complexity. The corvid family, in particular, includes some of the most socially complex species [40]. For instance, rooks (*Corvus frugilegus*) are pair-bonded birds that live in social groups throughout the year; multiple social groups merge into large colonies for breeding in spring and communal roosting in autumn but stay separate at other times of the year [40–44]. The social organization of rooks is thus highly complex, as individuals are involved in multiple layers of social connections in a frequently changing social environment where they may need to signal not only their own identity, but also what pair, social group, or even colony they belong to. We may therefore expect their repertoire to exhibit very high levels of diversity or flexibility that allow this identity signalling. At the group level, rooks possess a medium to large repertoire of calls [45,46], with evidence of an individual vocal signature in the most common ‘caw’ call unit produced by males [23]. Like several other corvids, they can also mimic other sounds, including anecdotal evidence of human voice mimicry [41,43,47] (N.S.C. and V.D., personal observations). Rooks also show long-term vocal recognition of conspecifics, have excellent learning, memory and planning skills, and more generally show good socio-cognitive skills [40,48,49]. They also appear to have the same song-related neural circuits as other oscines [50]. Aside from their calls, adult rooks, and some other corvids, also produce soft ‘songs’ [41,47,51]. These ‘songs’ are sequences of song units organized into phrases and can last from a few seconds to several minutes. However, unlike most other cases of birdsong, corvid ‘songs’ appear unrelated to either territorial defence or courtship [12,47], but instead resemble the undirected songs exhibited by several other songbird species [52–55]. This vocally atypical and socially complex species may therefore be a good model to evaluate vocal complexity in relation to cognitive and social complexity.

Our goal was to evaluate the complexity of vocal production in rooks. To do so, we recorded the vocalizations of two distant captive colonies, in Strasbourg (France) and Cambridge (UK). In contrast with previous studies, which rely only on standard acoustic parameters [23,56–59], we chose to construct each individual vocal repertoire through validated state-of-the-art machine-learning approaches [60,61]. We hypothesized that the high social complexity found in rooks should be associated with high vocal complexity, which should yield high diversity (large number of vocal units) and/or high gradation. Differences may be found between males and females, or between call units and song units. If vocal complexity is closely connected to the social relationship and structure existing in this species, we should find higher vocal similarity in call units between closely related individuals (pairs/colonies) compared to more distant individuals. In addition, if each individual rook produces complex and flexible songs as suggested in the literature [41], we should find low song unit similarity between individuals.

## 2. Material and methods

### 2.1. Study groups

The rooks in this study belonged to two captive colonies, one in Strasbourg (France) and one in Cambridge (UK). The individuals of each colony had been housed together since their capture as part of long-term research projects, except for one male in the Strasbourg colony. All birds except for this male had been caught as wild pre-flight juveniles and were hand-raised (see electronic supplementary material, table S1, for details, and table S2 for a tentative comparison with wild conditions). At the time of the study, 15 individuals (7 females, 8 males) comprised the Strasbourg colony and 7 individuals (4 females and 3 males) comprised the Cambridge colony. DNA testing had been used to ascertain the sex of each bird after their original capture. Finally, all birds were given coloured and/or numbered leg rings for identification purposes. Both colonies had food, water and enrichment available ad libitum, with food and water renewed daily.

In addition to the vocal complexity analysis in this study, we also analysed the social structure of the Strasbourg colony through proximity scans, to ascertain whether there were differences between certain individuals in social complexity (e.g. the total number and diversity of relations they had with other individuals). The results of this social analysis are shown in electronic supplementary material, figure S5.

### 2.2. Data collection

Both colonies were recorded and observed in several sessions with the same protocol. Autonomous recorders (Song Meter 4, Wildlife Acoustics, USA) recorded several hours each day. Each recorder bore two microphones on 3 m cables, and were spaced throughout the aviary to minimize distance from vocalizing birds (approximate maximum distance of 10 m). The dataset was thus composed of multichannel audio (2, 4 or 6 channels), digitized at 48 kHz with 16-bit resolution. Recordings from different recorders were manually synchronized before annotation.

Several recording sessions were attended by a human observer, throughout 2020 and 2021 for the Strasbourg colony and between February and April 2022 for the Cambridge colony. Vocalizations were noted in real time using a custom Python script, along with the identity of the emitter. Behavioural contexts for the vocalizations were determined by observation (based on [41]) and included when possible. Strasbourg sessions were taped using a video camera to help with the higher number of individuals. An expert observer then used these notes to annotate the recordings as spectrograms with the Audacity software (v3.1.3) [62]. We defined vocal units as vocalizations corresponding to continuous traces on the spectrogram, such as separating every element of a song sequence as song units, or as sequences of extremely short elements that only occurred together. We also made a distinction between two types of vocal units: songs (defined as series of at least 5 vocal units of at least 2 perceptually different types produced by one individual, with less than 10 s between successive vocal units), and calls (all other vocalizations).

This manually annotated dataset was used to train a deep neural network. This neural network is described in a previous paper [63]. In brief, this network replicates the manual annotation process (extracting start and end timestamps and individual identity for each vocal unit) using Mel-scale spectrograms corresponding to short chunks of audio. The network trained on the above dataset achieved approximately 72% retrieval of vocal units and 85% accuracy in identifying emitters in the retrieved vocal units. The same expert observer who annotated the previous dataset validated the network results by comparing them with the full audio recordings to ensure the same quality as the manual annotations, such that no false positive detections, false negative misses, or misidentifications remained when incorporating these results for the analysis.

The final dataset included 31 148 vocalizations from 47.9 h of audio recordings (29.1 h from the Strasbourg colony, 18.8 h from the Cambridge colony), of which 3 h from each colony came from unattended recording sessions.

### 2.3. Audio representation

The vocal units were extracted from the sound recordings and denoised with a dynamic spectral gating algorithm [61], then the microphone with the highest signal-to-noise ratio was selected. The audio was pre-emphasized and filtered, then converted to a spectrogram using a 10 ms Hamming window with 80% overlap. The spectrogram was then Mel-scaled to 80 frequency coefficients and constrained to above 100 Hz as a final noise-removing step. The spectrogram was trimmed to remove silent beginnings and ends, log-scaled, thresholded to a dynamic range of 20 dB below its maximum value, and finally normalized to between 0 and 1. This normalization removed most background noise and controlled for differences in amplitude and recording conditions between colonies and between different days [64]. Finally, only vocalizations from identified individuals that did not overlap other sounds were used in the analysis, keeping 25 220 vocalizations for further analysis.

### 2.4. Acoustic analysis

In this study, we sought to quantify and visualize the vocal complexity of rooks, which required the construction of a catalogue of the different vocal units produced by the species. We followed the procedure outlined here: define a measure of the acoustic distance between pairs of vocal units, compute the pairwise acoustic distances between the vocal units in the dataset, apply a dimensionality reduction algorithm followed by clustering to define groups of similar vocal unit, and finally use the clustering results to compute vocal complexity measures. Each step of this procedure is further detailed below and in the electronic supplementary material.

We considered vocal units to be similar if their spectrograms could be aligned in time and frequency. We selected a dynamic frequency–time warping (DFTW) distance to compute this alignment [65], although we used a constrained variant of the DFTW algorithm to reduce computational complexity (see electronic supplementary material). DFTW measures acoustic similarity between vocal units even if, for instance, their lengths or their frequency distribution differ. DFTW finds the optimal alignment between spectrograms by minimizing a cost function corresponding to the distance between the aligned spectrograms, and the value associated with this optimal alignment was used as the acoustic distance in our analysis.

The DFTW distance matrix, obtained from the acoustic distances between all pairs of vocal units in the data, contains the relative positions of the vocal units in the dataset with respect to one another. We used the UMAP dimensionality reduction algorithm [66] to project these positions to absolute coordinates in two-dimensional space for visualization. The projection was then used in clustering using the HDBSCAN algorithm [67,68] to group similar vocalizations together. HDBSCAN has the particularity of allowing soft clustering, where each vocal unit is not assigned to a single cluster, but instead to all clusters with varying probabilities. Both UMAP and HDBSCAN have become state-of-the-art algorithms due to their performance and robustness [69,70].

This analysis was carried out in Python (v3.8) using custom code for the DFTW, and the *umap-learn* (v0.5.3) [66] and *hdbscan* (v0.8.28) [67] packages for UMAP and HDBSCAN, respectively. Parameters were left at their default values in these packages, except for the following: UMAP *n_neighbors* = 30, *min_dist* = 0; HDBSCAN *min_samples* = 30, *min_cluster_size* = 30. UMAP parameters were validated by checking that the UMAP projection preserved the structure of the dataset with the trustworthiness and continuity measures [71]. Trustworthiness was 0.96 and continuity was 0.92, where 1 represents perfect preservation, so we proceeded with the analysis. HDBSCAN parameters were chosen to avoid small clusters that may emerge purely by chance.

### 2.5. Quantifying repertoire complexity

We used the soft clustering results to quantify three aspects of complexity: repertoire diversity (the number of different vocal units in the repertoire of an individual), repertoire gradation (a measure of separation between categories of vocal units; gradation is low if categories are well-separated with no intermediate vocal units), and inter-individual repertoire similarity (the extent to which two individuals share their repertoires).

We quantified diversity and gradation using two information theory indices, Shannon's diversity index and Simpson's diversity index [72] (see also electronic supplementary material), which represent the effective number of same-size clusters in the results from HDBSCAN. Shannon's diversity weighs all clusters equally, while Simpson's diversity gives more importance to large clusters. These indices can take values between 1 and the total number of clusters. We normalized the values to between 0 and 1 to make them comparable with future results, so that a vocal unit attributed to only one cluster corresponded to a value of 0, and a vocal unit attributed to all clusters with equal probability corresponded to a value of 1. For repertoire gradation, we computed one index value per vocal unit. For repertoire diversity, we computed one index value per individual by averaging the soft clustering results of all vocal units produced by this individual. We note that this may cause a strong correlation between the two indices, since we compute them from essentially the same data. In practice, this stresses the importance of using not just one or the other, but both indices, to avoid ambiguities (e.g. mistaking a highly graded repertoire for a highly diverse repertoire) by looking at the data through multiple perspectives.

For inter-individual similarity, we used the Morisita–Horn overlap index [73] (see also electronic supplementary material), which represents the probability that two vocal units, one each randomly drawn from the repertoire of different individuals, fall in the same cluster. The index is bounded between 0 and 1, with 0 representing complete dissimilarity (no common clusters between the individuals) and 1 representing complete similarity (both individuals produce vocalizations from the same clusters with the same distribution). We computed the index for each pair of individuals by averaging the soft clustering results of all vocal units produced by each individual.

### 2.6. Statistical analysis

Statistical analysis of the distribution of gradation, diversity and similarity was carried out using generalized linear models (GLMs) or generalized linear mixed models (GLMMs). Models were fitted in R (v4.1.1) [74] with the *glmmTMB* package (v1.1.7) [75]. We chose ordered beta regression as the response variables were the indices, represented as continuous variables bounded between 0 and 1 [76]. We built full models with sex, vocal unit type and colony as predictors, as well as all second-order interactions. For diversity and gradation, individual identity was also included as a random factor. For inter-individual similarity, the sex and vocal unit type included comparisons within and between levels (e.g. for sex, we compared males with other males, females with other females and females with males); the colony variable only compared within the same colony or between different colony (we did not distinguish between the Strasbourg and Cambridge colonies due to the lower number of singing birds in the latter). We then performed model selection with the *MuMIn* package (v1.43.17) [77], fitting all models obtainable by removing variables from the original full model (including the null model by removing all variables), and ranking them by increasing AICc. In this procedure, the best model has the lowest AICc, and any model more than 2 AICc units above this model is significantly worse at predicting the data [78]. The quality of the selected best model was then assessed visually through QQ plots of the model residuals against quantiles of a normal distribution with mean 0 and variance 1. We finally performed pairwise tests on the best model with the *emmeans* package (v1.8.0) [79], and Tukey's method was used to correct *p*-values for multiple testing.

## 3. Results

### 3.1. Dataset distribution

Vocalization rates (vocal units produced per individual per hour recorded) were lower for females than males in both colonies, and were also lower for the Cambridge colony in general compared to the Strasbourg colony (electronic supplementary material, table S3). This meant that some females were sampled far less (in particular, one female from the Cambridge colony accounted for less than 200 vocal units). The dataset was split approximately equally between call (45.9%) and song (54.1%) units. Both males and females produced song.

Contexts for the vocal units were determined from observing the behaviour of the emitter at the time of production. Only 33% of vocal units in the dataset could be contextualized in this manner, with equal repartition between males (52% of contextualized vocal units) and females (48%). The specific contexts associated with these vocal units differed between sexes. Contextualized vocal units from females were most often associated with a bow and tail fanning posture (27% of these vocal units), nest building (26%), receiving food (20%) and nest calling (10%). Contextualized vocal units from males were most often associated with a bow and tail fanning posture (58% of these vocal units), play or object exploration (21%) and foraging (11%). Furthermore, 53% of contextualized vocal units correspond to song, with 85% produced by males. Among these song units, 9% were also associated with a bow and tail fanning posture, 7% with play or object exploration, 1% with a copulatory display and under 0.1% with mounts, foraging and feeding another bird.

### 3.2. Projection and clustering results

**Figure 1.**
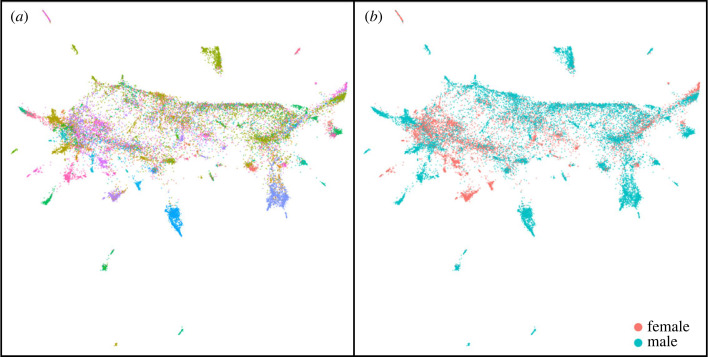
UMAP embeddings of the vocal units in the data, with different colour schemes: (*a*) individual identity (legend omitted to reduce clutter) and (*b*) sex. See electronic supplementary material, figure S2, for projections also coloured by colony and vocal unit type.

All vocal units were projected into two-dimensional space with UMAP, showing several well-defined clusters around a more diffuse region (figure 1). Many clusters appeared homogeneous, including only one individual and either call units or song units. HDBSCAN found 80 clusters, accounting for 68.3% of the vocal units. The remaining 31.7% of vocal units were too sparse to assign to a cluster. To avoid dropping them from the analysis, we used the soft clustering results, where each vocal unit is assigned to all clusters with varying probabilities.

### 3.3. Repertoire gradation

We quantified repertoire gradation with Shannon's diversity and Simpson's diversity. Both indices resulted in similar statistical results; we therefore solely discuss Shannon's diversity here (see electronic supplementary material, table S4 and figure S3, for the same analysis with Simpson's diversity). The best model included only sex, vocal unit type and the interaction between them. The null model was significantly worse at predicting repertoire gradation (425.4 AICc units above the best model).

**Figure 2.**
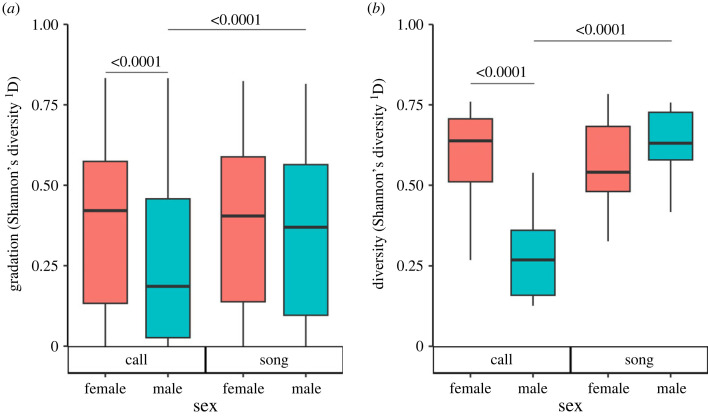
Analysis of (*a*) repertoire gradation and (*b*) repertoire diversity by sex and vocal unit type, measured by Shannon's diversity. The two colonies were not statistically different and so were pooled together. Horizontal lines represent pairwise tests with the associated *p*-value above. For clarity, only tests where one variable varies are shown (e.g. comparing call units between males and females, or comparing between call units and song units in males, but not between male call units and female song units).

Pairwise tests (figure 2*a*) showed that male call units had significantly lower gradation than either male song units (odds ratio, call units to song units: 0.70 ± 0.01, *z* = −20.30, *p* *<* 0.0001) or female call units (odds ratio, females to males: 1.87 ± 0.20, *z* = 5.90, *p* *<* 0.0001). By contrast, female call units were not less graded than female song units (odds ratio, call units to song units: 1.08 ± 0.03, *z* = 2.40, *p* = 0.08), and male song units were not less graded than female song units (odds ratio, female to male: 1.20 ± 0.13, *z* = 1.72, *p* = 0.31).

### 3.4. Repertoire diversity

Like with repertoire gradation, similar statistical results for repertoire diversity were obtained with both Shannon's diversity and Simpson's diversity indices; we therefore only discuss Shannon's diversity here (see electronic supplementary material, table S4 and figure S3, for the analysis using Simpson's diversity). The best model included only sex, vocal unit type and the interaction between them. The null model was significantly worse at predicting repertoire diversity (25.6 AICc units above the best model).

Pairwise tests (figure 2*b*) showed that male call units were significantly less diverse than either male song units (odds ratio, call units to song units: 0.23 ± 0.04, *z* = −7.81, *p* < 0.0001) or female call units (odds ratio, females to males: 4.01 ± 1.01, *z* = 5.51, *p* < 0.0001). By contrast, male song units were no more diverse than female song units (odds ratio, females to males: 0.82 ± 0.24, *z* = −0.67, *p* = 0.91), and female call units were no more diverse than female song units (odds ratio, call units to song units: 1.12 ± 0.24, *z* = 0.57, *p* = 0.94).

### 3.5. Inter-individual repertoire similarity

**Figure 3.**
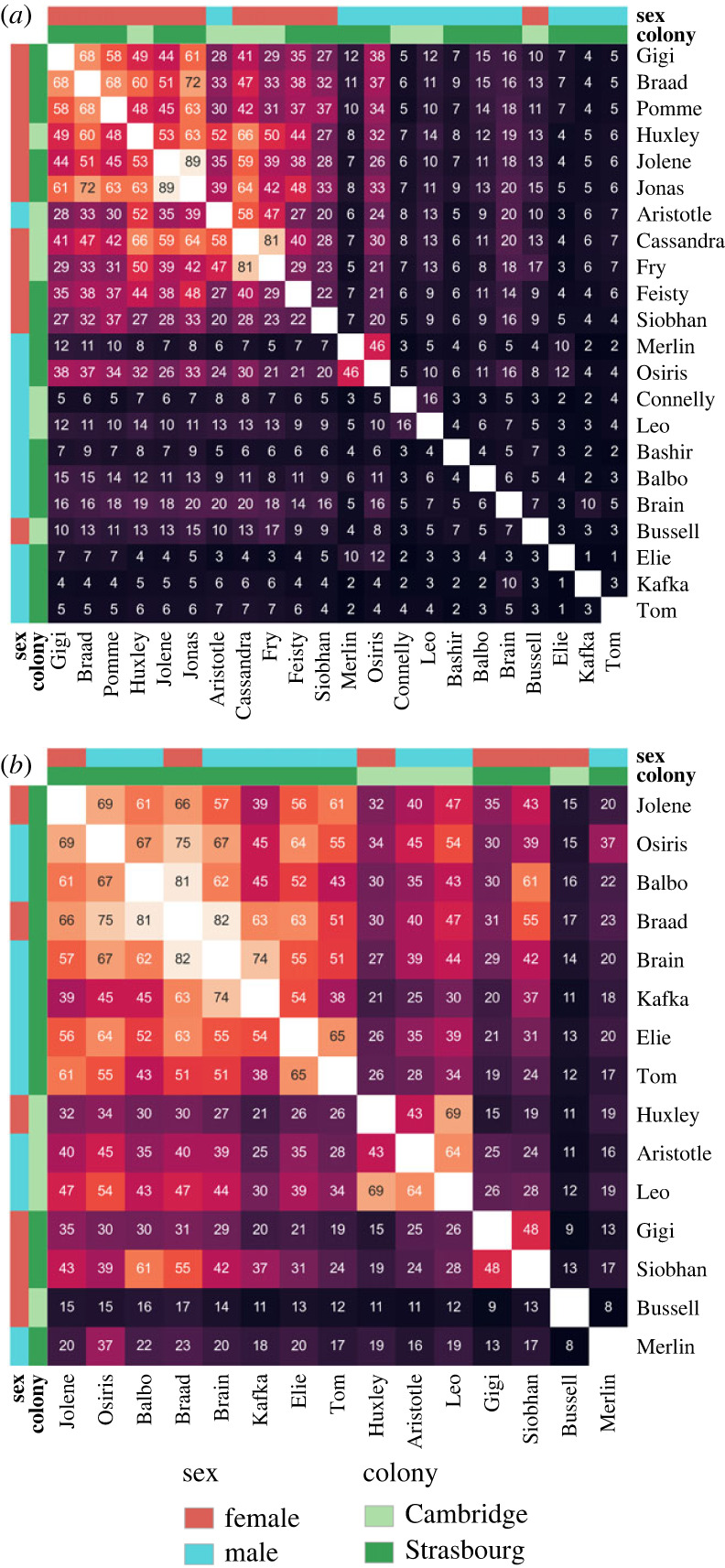
Pairwise inter-individual repertoire similarity, measured by the Morisita–Horn overlap index. Each heatmap corresponds to a similarity matrix, where each row and column represents one individual. (*a*) For call units. (*b*) For song units. Each matrix was reordered through hierarchical clustering so individuals were grouped by their pattern of similarity with respect to other individuals. Light cells indicate high similarity, with the associated index value inside the cell in percent.

We assessed inter-individual similarity with the Morisita–Horn overlap index, which quantifies to what extent the vocalizations of two individuals fall in the same clusters (figure 3). The best model was the full model (electronic supplementary material, table S4). The null model was significantly worse at predicting inter-individual repertoire similarity (296.4 AICc units above the best model).

**Figure 4.**
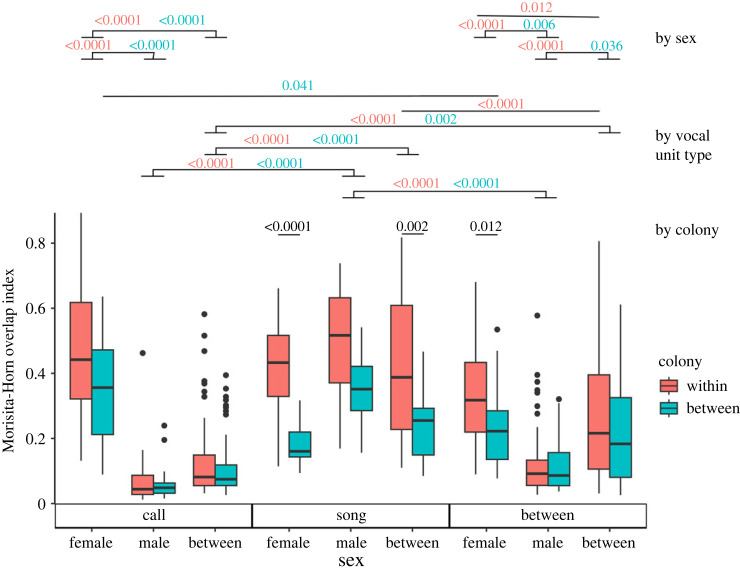
Analysis of inter-individual repertoire similarity by sex, vocal unit type, and colony, measured by the Morisita–Horn overlap index. Boxplots indicate distributions of similarity values. Horizontal lines represent pairwise tests with the associated *p*-value above. Analogous comparisons within and between colonies, when they are associated with the same result (e.g. top left, second comparison from the top: the comparison of male call units versus female call units are significant both within the same colony and between colonies, so they are represented on the same line, with the *p*-value associated with the ‘within colony’ test in red and the *p*-value associated with the ‘between colony’ test in blue). For clarity, only tests where one variable varies are shown and grouped by which variable is being compared (for instance, the bottom three comparisons are ‘by colony’, i.e. comparisons between individuals 'within' the same colony, or between individuals 'between' different colonies, but involving the same vocal units, e.g. female song units for the leftmost of these comparisons).

The most striking result concerned the extremely low inter-individual similarities in the call units of males (figure 4), even within the same colony. Male call unit repertoire similarity was significantly lower than either female call unit similarity (odds ratio, females to males within the same colony: 8.04 ± 1.39, *z* = 12.03, *p* < 0.0001) or male song unit similarity (odds ratio, call units to song units within the same colony: 0.11 ± 0.02, *z* = −11.89, *p* < 0.0001). By contrast, females were far more similar in their call unit repertoires, even between different colonies (odds ratio, female to male between different colonies: 4.82 ± 0.85, *z* = 8.87, *p* < 0.0001). This was a very consistent pattern, with very few exceptions (figure 3*a*). For instance, two Strasbourg males were more similar to each other (Merlin and Osiris, 46% overlap index), and one Cambridge female was highly dissimilar to all other individuals in either colony (Bussell, 317% overlap index). Moreover, while many individuals in both colonies were in breeding pairs (electronic supplementary material, table S1), there was low call unit similarity between males and females in general (figure 3*a*, figure 4 leftmost panel), which precluded similarity between the members of a pair. To illustrate these results, examples of the most frequently uttered call units from every individual can be found in the electronic supplementary material (see also electronic supplementary material, figure S6). Unlike call units, song unit repertoire similarity did not differ between sexes (odds ratio, females to males within the same colony: 0.80 ± 0.18, z = −0.95, *p* = 1; females to males between different colonies: 0.48 ± 0.11, *z* = −3.07, *p* = 0.17). Most individuals had intermediate to high song unit repertoire similarity, even between colonies (figure 3*b*).

Comparing between song units and call units (i.e. whether the same vocal units may be used both during song and as call units), the similarity matrix (electronic supplementary material, figure S4) suggested that males, despite higher song unit repertoire similarity, did not incorporate call units in their song unit repertoire. Females were significantly more similar to one another in this respect (odds ratio, females to males within the same colony: 3.21 ± 0.47, *z* = 7.90, *p* < 0.001; between different colonies: 1.92 ± 0.31, *z* = 4.07, *p* = 0.006). These results excluded self-similarities (i.e. between the call and song units of the same individual); nonetheless, self-similarities showed no apparent pattern: some individuals were highly self-similar (e.g. Aristotle 76%, Bussell 98%, Braad 85%, Merlin 93%), others much less so (e.g. Tom 6%, Kafka 17%), with no effect of sex or colony.

Despite being included in the best model, few pairwise tests on the effect of the colony were significant. Only three tests showed significantly higher similarity within the same colony (figure 4, black *p*-values): in female song units (odds ratio, within the same colony to between different colonies: 2.69 ± 0.50, *z* = 5.37, *p* < 0.0001), between female call units and female song units (odds ratio, within to between: 1.72 ± 0.24, *z* = 3.88, *p* = 0.012), and between male song units and female song units (odds ratio, within to between; 1.93 ± 0.29, *z* = 4.37, *p* = 0.002). Otherwise, there was no difference in similarity when comparing individuals from the same colony and individuals from different colonies.

## 4. Discussion

We evaluated the vocal complexity of the calls and songs of two colonies of rooks, a species with high social complexity and cognitive ability, through the analysis of the diversity, gradation and inter-individual similarity in their vocal repertoire. Male call units had the lowest gradation and diversity, while female song units, female call units, and male song units were not significantly different in either gradation or diversity. Thus, we detected vocal complexity but not necessarily where we had hypothesized. Indeed, we expected vocal complexity to mirror social complexity, through call or song similarity at the pair and colony levels. In the majority of vocal species, a vocal repertoire is composed in majority by clusters reflecting different call types, with some degree of gradation potentially present, but we found large degrees of gradation that precluded segregation into call types, especially for female call units. Finally, the vast majority of call units in this study could not be conclusively associated with specific behaviour contexts, which is consistent with other literature on corvid vocal communication [45–47]. However, one particular call type could be conclusively associated with a behavioural context: the nest call, produced only by brooding females. This association between acoustic structure and behavioural context was confirmed in our analysis, as almost all nest calls were projected close to other nest calls in the UMAP projection (see electronic supplementary material, figure S7). We note that this study is the first to quantify vocal complexity in corvids, although a fairly similar study has been conducted in macaques [80]. Nevertheless, these results show high vocal complexity via diversity, gradation and functional uncertainty in the repertoire [7], which still may reflect some elements of social complexity in the life of rooks.

Our most striking result, and directly contrary to our hypotheses, is the observation that call unit repertoires of individual males were all almost completely dissimilar even within the same colony, unlike the highly similar call unit repertoires shared by females even between different colonies. Interestingly, most of the female calls that could be contextualized were either food-related or associated with a breeding context (e.g. nest calls emitted by brooding females). These calls may be more biologically significant in females, who depend entirely on their mates feeding them during much of the breeding season [81,82]. As such, nest calls may be under stronger pressure for inter-individual homogeneity within and between individuals and groups. These essential communicative functions may explain why the call units of the females are more similar than those of the males. Unlike females, males in both colonies exhibited highly individualized call unit repertoires. Our study is the first to quantify this individual heterogeneity using objective measurements, expanding on the previous literature on the repertoire of rooks [23,45] and other corvids such as American crows [22], and Hawaiian crows [83], where this heterogeneity was suspected but could not be quantified. In rooks, the most frequently uttered ‘caw’ call unit has been reported to vary greatly between individuals, especially in its duration [23,45], while two other call units, the ‘gull’ and ‘squalling’ calls, varied in frequency modulation [45]. However, previous repertoires grouped calls into functional categories rather than by acoustic structure. In most bird species, individuals produce similar call repertoires [2], which arise through vocal learning during development [81,84,85] or through vocal convergence between social partners [32,86–88]. Inter-individual heterogeneity has been reported in very few cases, such as in the contact calls of parrots [25,89], the signature whistles of bottlenose dolphins [35], or the food begging calls of juvenile chipping sparrows [90]. However, it is not always clear if the reported differences are due to heterogeneity as we observe in this study or to individual vocal signatures [21], and none of these previous results were at the scale of the entire call repertoire. The large acoustic heterogeneity detected in male rooks suggests that they are weakly influenced by vocal productions of others, and thus reduces the probability of detecting group signatures or dialects in their calls. This heterogeneity, and the lower diversity found in male call units, could not be linked to lower social complexity in males compared to females (see electronic supplementary material, figure S5). While analogous data are currently lacking in wild rooks, personal observations and the existence of the same patterns in two independent captive colonies mean that our results are unlikely to be artefacts of either captivity or atypical vocal behaviour. In particular, rooks are capable of vocal mimicry of others and environmental noises [41,43,47], thus inter-male heterogeneity could have been explained by mimicry of and in different environments. However, we found no evidence that individuals from the same colony at the time of the study, or individuals captured from the same colony (see electronic supplementary material, table S1), were more similar, as could have arisen from social exposure to conspecifics, or common mimicry of environmental sounds at the respective locations of the colonies. In addition, female call units were remarkably similar between colonies despite the geographical distance between the colonies. The high inter-individual heterogeneity in male rook calls raises the question of how they communicate within the group. Communication *a priori* requires common vocalizations for adequate information exchange, which was not the case in males here. One alternative is that rooks may memorize the calls used by each male in a given context. Corvids are among the only nonhuman species to have demonstrated episodic-like memory, the ability to recall not only what an event was, but also where and when it occurred [91–93], and so might have the ability to memorize the repertoires of other individuals.

Unlike call units, song unit repertoires were more similar between individuals, and might thus be better indicators of colonial origin. However, we again found no support for this hypothesis, as there was no clear dissimilarity between colonies. Despite this, rook song appears atypical compared to other birdsong. Notably, both sexes sang with similar degrees of unit diversity and gradation, indicating similar song complexity, and in circumstances unrelated to territorial defence or courtship. In fact, rooks often actively seek to perch alone to sing, and sing very softly compared to their calls [41]. For instance, in our study, a singer was often only heard on the microphones closest to its position in the aviary, but not on microphones only a few metres away. In general, corvid song has rarely been studied in the literature beyond passing mentions [41,47,51], and female birdsong has historically been understudied compared to male song [94]. What does exist questions the function of rook songs compared to what is most often described in the literature [1]. One possibility is that rooks may actively seek vocal individuality in order to remain distinct in frequently changing social environments, such as when social groups merge into larger colonies [41]. Vocal avoidance could then promote vocal innovation, itself encouraged by regular song practice, and in turn explain both high call heterogeneity and high song gradation and diversity. Nevertheless, this unusual manner and context for singing is an integral part of the vocal repertoire, and thus contributes to the vocal complexity of this species. Further studies are needed to evaluate individual innovation by looking at individual patterns or rhythm and their stability in the songs of this species.

High inter-individual heterogeneity may exist in other species, but may have remained undetected due to methodological limitations. The computational capability to compare large datasets of complete representations of vocal units, such as entire spectrograms, in a reasonable timeframe has emerged only recently [60,61,65,95], and previous studies usually used either human classification or specific acoustic parameters, such as spectral or temporal energy, fundamental frequency or frequency contour trajectory [61]. The methodology used here is based on this principle of complete representation. As such, inter-individual differences can be highlighted, without many of the constraints in acoustic parameter-based approaches. For instance, individual vocal signatures can be controlled for if they are based only on frequency or time differences (e.g. pitch or duration; see electronic supplementary material, figure S7). This approach allows fine-grained analysis of vocal units for studying vocal complexity in any animal species, representing a highly effective tool, in both objectivity (by largely removing human-based decisions in choice of parameters or classification of vocal units, whether functional or structural) and scalability (by limiting the time needed to examine a corpus of data to classify vocal units). We have demonstrated its use to measure not only repertoire diversity and gradation but also inter-individual similarity, which we suggest should also be included as a component of vocal complexity.

To our knowledge, this study is the first to focus on constructing the vocal repertoire of a corvid species based on individually identified birds. Moreover, our findings are all the more robust as they are based on two independent colonies that had never been in contact. Further studies are now needed to determine the main factors (ecological, cognitive, or social) that promote this vocal complexity. Approaches such as the one used here prove powerful and robust, and may be applied to other bird species with high vocal complexity, such as parrots [10,86], the nightingale *Luscinia megarhynchos* [96], the brown thrasher *Toxostoma rufum* [97] the lyrebird *Menura alberti* [98] or the mynah *Gracula religiosa* [99]. More generally, contrasting the vocal repertoire and flexibility of species with varying social organization complexity and cognitive performances should provide insights into the determinants of vocal complexity and its links with social complexity in various animal species.

## Data Availability

The data used in this study (audio and annotations) are available at https://zenodo.org/records/8036310. The code is available at https://gitlab.com/kimartin/cluster_rook_vocs. Supplementary material is available online [100].
